# Meaning-Making Narratives Within a Puzzle of Parts: A Psychobiographical Sketch of Sylvia Plath

**DOI:** 10.5964/ejop.5321

**Published:** 2021-08-31

**Authors:** Angela F. Panelatti, Joseph G. Ponterotto, Paul J. P. Fouché

**Affiliations:** 1 Department of Education, Eunice High School, Bloemfontein, South Africa; 2 Graduate School of Education, Fordham University, New York, NY, USA; 3 Department of Psychology, Free State University, Bloemfontein, South Africa; Nelson Mandela University, Port Elizabeth, South Africa

**Keywords:** Sylvia Plath, psychobiography, Internal Family System model, polarisation of parts, creative meaning-making narratives

## Abstract

This study aimed to unveil Sylvia Plath’s (1932–1963) meaning-making narratives, within her life’s puzzle of parts, by utilising the Internal Family System (IFS) model of Schwartz. Plath was purposively selected as subject since she has been proclaimed as one of the most renowned and influential voices in 20th century Anglo-American culture and literature. Although she only published one collection of poems, “The Collosus”, and one novel, “The Bell Jar”, in her lifetime, the plethora of short stories, poems, journal entries and letters which were published after her suicide secured her status as a powerful and creative voice. Methodological strategies utilised to sort and integrate the wealth of publically-available socio-historical data on Plath included the analysis of psychobiographical indicators of salience according to the model of Irving Alexander and the data analysis matrix procedure of Robert Yin. Findings suggest that each stage of Plath’s life was characterised by “parts-led” functioning as a result of transferred burdens, imperfect care-taking, existential anxiety and traumatic emotional experiences. This resulted in polarisation of her different parts, which blocked the healing energy of her Self and aggravated feelings of worthlessness, in spite of her creative meaning-making narratives. Since Sylvia used her creative genius to address socio-historical issues and injustices, her life lends itself to meaning-making narratives, especially those that empower and inspire future generations of previously disempowered groups.

Over the years the picture that I have built up of my mother is of a woman with good and bad parts; huge talent and emotional difficulties, huge love and an aptitude for jealousy—just like many of us. We are all a balance of the parts that make us, and it is up to us which of those parts we allow most emphasis. My mother fought with the negative parts of her makeup, and, even though she lost her battle in the end, she used the material of it in her poetry. (Sylvia Plath’s daughter, Frieda Hughes, in her postscript to the restored *Ariel* edition by Sylvia Plath, 2004, pp. 6–7)

Sylvia Plath (1932–1963) has been proclaimed as one of the most renowned and influential voices in 20th century Anglo-American culture and literature (Gill, 2008; Perloff, 1979). Although she only published one collection of poems (*The Colossus*) and one novel (*The Bell Jar*) in her lifetime, the plethora of short stories, poems, journal entries and letters which were published after her suicide not only secured her status as a powerful feminist voice, but also added to the haunting allure of her life, which was shrouded in a web of controversies (Gill, 2008; Kumlu, 2011; Perloff, 1979; Rose, 2013). These controversies were compounded by the fact that Sylvia was still married to Ted Hughes when she died, resulting in the Plath Estate falling into the hands of Ted and his sister, Olwyn. They not only discouraged any form of research and interpretation, but apparently even threatened to take legal action against biographers who were unwilling to subordinate themselves to the demands and prerequisites of the Plath Estate (Alexander, 1999; Butscher, 2003; Gill, 2008; Rollyson, 2013). Despite the plethora of literary critiques, memoirs and biographical interpretations, Sylvia Plath’s life had never been the subject of any previously-conducted psychologically-informed biography—a fact which provided the motivation behind this study since it hoped to illuminate the psychological aspects hidden behind the mystery of this literary genius.

The myths that have been created concerning Sylvia Plath, especially those starting from her death, are not only the false assumptions of literary critics, but also of the Cold War American ideology, which tried to turn Plath into a problematic woman (Gill, 2008; Kumlu, 2011; Perloff, 1979). Despite the claims of Sylvia Plath’s critics, that her works were built mainly upon a life story characterised by despair, Kumlu (2011) argued that the real Plath succeeded in crystallising not only the traumas of her generation, but also various literary works as a response to the ideology of her age. This view corresponds with that of Gloria Steinem, one of Sylvia Plath’s classmates (Alexander, 1999), and a leading spokeswoman for the American feminist movement in the late 1960s and early 1970s. Steinem described Plath as being an early prophet who used her suffering to describe societal problems (Alexander, 1999). In terms of this description, Plath’s works not only encompass pessimism, but also happiness, achievement and power—a view seldom taken of Plath’s life and works.

The Internal Family System (IFS) model of Schwartz (1995) assumes a non-pathologising approach and offers a more hopeful and positive framework compared to the psychopathological and psychoanalytical stances often taken in psychobiographical studies. It was chosen as a psychological framework to investigate and delineate Sylvia Plath’s personality development precisely because the authors wanted to focus on the positive, more hopeful aspects of her creative genius, rather than on the pathologising approaches which have typically been elicited from her life and works.

One recent observation of the psychobiographical field, is that the body of historical subjects studied has been primarily male, and modern psychobiography advocates for increased attention to female personalities (Young & Collins, 2018). Apart from contributing to the body of knowledge on Sylvia Plath and to the framework of the IFSs model, this study affirmed that an examination of the lives of extraordinary women who used their creative genius to address socio-historical issues, could be a significant endeavour for future psychobiographical researchers. Whilst it is true that Plath suffered from depression, and that many of her works encompass pessimism, to label someone of Plath’s calibre as schizophrenic or mad not only undermines the value of her contribution to literature and to the feminist movement, it also disregards the complexity of her nature and the impact of her socio-historical context on her development.

## Method

### The Psychobiographical Subject

Sylvia Plath was selected as a psychobiographical subject by means of purposive sampling. Her life, which spanned 30 years from the time of her birth on 27 October 1932, till the time of her death on 11 February 1963, was explored and described against the backdrop of the socio-historical contexts in which her writing was produced. Information on the life of Plath was gathered by means of collecting publically-available biographical, autobiographical and literary material on Plath, as well as material related to the historical period in which she lived. The information gathered on Plath was organised into three major phases of development: infancy and childhood, adolescence, and adulthood.

### Theory Selection

The literature review indicated that Plath’s life and personality development appeared to have theoretical value and applicability to the psychological framework which was chosen for this study. Schwartz’s IFS model evolved out of the recognition of the multiplicity of the mind and an attempt to understand it using systems thinking (Schwartz, 1995, 2001). The IFS model emphasises the importance of viewing the individual as a system composed of various subpersonalities or parts (Schwartz, 2003; refer to Table 1).

**Table 1:** Components of the Internal Family Structure

**The Self** The compassionate, inner leader containing the confidence and vision to lead an individual’s internal and external lives harmoniously.					
**Exiles** Young parts that have experienced trauma and become isolated from the rest of the system in an effort to protect the individual from feeling the pain of these parts.					
Protectors					
**Managers** Parts that run the day-to-day life of the individual. Attempt to keep the individual in control in every situation and relationship in an effort to protect parts from feeling any hurt or rejection.					**Firefighters** Parts that react when exiles are activated in an effort to control and extinguish their feelings. They can do this in a number of ways, including: Drug or alcohol-use, self-mutilation, excessive behaviour.
**Striver** (Excessively task-orientated, controlling and critical)	**Evaluator** (Perfectionistic, wants social approval)	**Passive Pessimist** (Apathetic, avoids personal risk)	**Caretaker** (Selfless and caring) **Entitled One** (Selfish and self-serving)	**Worrier or Sentry** (Anxious, alert for danger)	

At the core of all these parts is the Self, which can be defined as an active, compassionate inner leader (Haddock, Weiler, Trump, & Henry, 2017; Schwartz, 1987, 2001, 2013a). In line with the approaches advocated by family therapy, the IFS model proposes that individual improvement is attained not through the removal of a dysfunctional part, but rather through the acceptance that each part is significant and valuable (Green, 2008). The internal parts assume characteristics which maintain functionality (managers), harbour pain (exiles), or react to impending threats (firefighters) (Twombly & Schwartz, 2008).

The optimistic philosophy inherent in the IFS model allows for people to be seen as having all the resources that they need for healing to take place (Schwartz, 1995). Rather than seeing people as having pathological deficits, the IFS model sees people as being restricted from using internal strengths as a result of polarised relationships both within themselves and in relation to people around them (Earley, 2012; Schwartz, 1995, 2013b; Sweezy & Ziskind, 2013). According to Schwartz (1995), polarization occurs when a system is imbalanced. When polarization occurs, parts become antagonistic as each member of the polarization clings rigidly to his or her position, fearing that the system will be damaged if that rigidly held position is relinquished (Watzlawick, Weakland, & Fisch, 1974).

### Data Extraction and Analysis

Alexander’s (1990) indicators of salience were used as a guide in the identification of psychologically significant incidents, experiences or themes pertaining to the life of Plath. A data analysis matrix (Yin, 2009) facilitated the analysis of psychobiographical data as modelled in Fouché (1999). During the collection, examination, condensation, extraction and analysis of salient biographical material pertaining to Plath’s IFS, particular attention was given to the sub-personalities which emerged as distinct parts (Goulding & Schwartz, 1995) as Plath passed through significant periods in her life. The impact of life-changing forces and historical events on the expression of the sub-personalities throughout Plath’s life was also taken into consideration. (Additional information on Alexander’s indicators of salience and the data analysis matrix are available upon request from the senior author.)

### Trustworthiness and Ethical Considerations

With regard to trustworthiness, methodological triangulation included both data source triangulation and researcher triangulation (team of three researchers). Data on Sylvia Plath was sourced solely from publicly-available material, thereby minimising the possibility that any of Plath’s surviving family members, friends or colleagues could be harmed or embarrassed by revelations which unpublished controversial information could cause (Ponterotto & Reynolds, 2017). Furthermore, Plath’s functioning and development were examined both from a longitudinal and eugraphic perspective, allowing for her life story to be viewed not only in terms of suffering, despair and trauma, but also in terms of happiness, achievement and power (a view seldom taken of Plath’s life). The research was approved by the Institutional Review Board of the Faculty and the Committee for Title Registrations of the University of the Free State (UFS).

## Findings and Discussion

### The Activation of the Protectors during Infancy and the Childhood Years (1932–1944)

Sylvia Plath was the first-born child of Otto Emile Plath and Aurelia Schober and her first year of life coincided with the Great Depression, which followed the stock market crash of 1929 (Alexander, 1999; Butscher, 2003; Wilson, 2013). After Otto’s death, the Plath family found itself in serious financial difficulties, due to the fact that Otto had lost a substantial amount of money on the stock market (Alexander, 1999; Butscher, 2003; Plath, 2000). The family’s financially precarious position had a profound impact on Sylvia and contributed significantly to her life-long sense of inferiority from the perspective of Schwartz’s (1995, 2001) model. Furthermore, Otto’s tyrannical disposition and authoritarian demand for order and Aurelia’s submissive obeisance to his autocratic method of ruling his household, are speculated to have created an imbalanced system, resulting in polarisations of Sylvia’s internal and external family system, and the activation of what Schwartz (1995, 2001) referred to as protectors (Managers and Firefighters).

Sylvia’s lifespan between 1 and 4 years coincided with the birth of her brother, Warren, and the family’s move to Winthrop, a city in Massachusetts, in the United States. Both these events constituted significant occurrences which impacted profoundly on Sylvia’s development and functioning according to Schwartz’s (1995, 2001) model. Warren’s birth resulted in Sylvia’s internal family parts acquiring burdens as a result of attachment disruptions, which she experienced as traumatic and which made her feel unloved and worthless. The trauma aggravated Sylvia’s polarised system, resulting in a fragmented system of antagonistic parts and a life-long rivalry with Warren. This rivalry extended into Sylvia’s adulthood and came to symbolise the larger battle that she had to fight with men throughout her life in order to gain recognition as an independent, successful woman (Panelatti, 2018).

Sylvia’s increasing sense of insecurity prompted her to establish a stronger attachment to her father, whom she sought to please and impress by means of her intellectual abilities. In their desperation to be saved, her exiles idealised her father, whom she described as “a giant of a man” who “feared nothing” (Plath, 2008, p. 320). Sylvia’s tenacious persistence to learn the alphabet and her subsequent attachment to words as a substitute for love, attest to the powerful influence of her critical, controlling Striver. In fact, Sylvia identified with the physicality of words to such an extent that she often wished, as she expressed in *The Bell Jar*, that she could return to the “womblike space of the printed page” (Plath, 1966, p. 57). Her Striver pushed her to strive for literary excellence so that she could be distracted from exiled feelings of worthlessness that might cause pain. The powerful influence of Sylvia’s Striver manifested in her life-long, obsessive pursuit of literary achievement, and although this obsession haunted her, it also spurred her on in her search for meaning. Her attachment to words was reinforced by her belief that perfecting her linguistic capabilities would guarantee her parents’ love and ensure that they never abandoned her (Panelatti, 2018).

Sylvia’s tendency of escaping to an exotic, fantasy world attests to the emergence of her dissociating parts. Her firefighters prompted her to escape to a fantastical “never-never land of magic” (Plath, 2000, p. 35) to protect her from the painful reality of her exiled parts. Sylvia’s childhood tendency of escaping to the magical, fantasy-world which she created for herself through her literature, continued into adulthood and served as a powerful tool in her search for meaning. Although an illusion, that fantasy world held the promise of a meaningful literary legacy—one that would inspire the meaning-making process for future generations (Panelatti, 2018).

In 1936, during middle childhood and a year after Warren’s birth, Sylvia’s father Otto began to suffer from ill health (Wilson, 2013). Although he was an irritable, short-tempered man who was often consumed by “explosive outbursts of anger” (Plath, 1992, p. 18), Sylvia’s vivid artistic imagination ensured what she described as “an idyllic childhood” (Wilson, 2013, p. 31). Unfortunately, the ever-worsening progression of Otto’s ill health exacerbated his already volatile temperament, and constituted a significant occurrence which had a profound impact on Sylvia’s development. Otto’s temper worsened to the point that Aurelia took Sylvia and Warren to live with her parents. Some months later, they moved to a more spacious house at the coast in Winthrop. The move constituted a major event in Sylvia’s life, since it was in Winthrop that she acquired a powerful, almost obsessive love of the ocean, one which fuelled her poetic imagination.

World War ΙΙ occurred during Sylvia's pre-adolescent years, and she was particularly susceptible to the negative repercussions of anti-German sentiment at this time. Not only did the war affect Sylvia’s development and shape her views of herself and the world, it also promoted her artistic imagination and intensified her academic zealousness (Alexander, 1999; Butscher, 2003; Kirk, 2009).

She maintained an outstanding academic profile, exhibiting superior intelligence and extreme dedication throughout her schooling years. Despite remarkable accomplishments, however, her journal entries indicate that she was eternally dissatisfied with herself. Sylvia’s critical Striver impelled her to strive for outstanding achievement to distract her both from her own personal feelings of unworthiness, and from the legacy burdens transferred to her by her own rigid and controlling parents (Panelatti, 2018).

Events leading up to and including the death of her father in 1940, constituted a major traumatic event in Sylvia’s life. This trauma was intensified because she experienced his departure as a deliberate act of betrayal (Butscher, 2003). Sylvia had come to depend upon her father’s praise (Wilson, 2013). Consequently, she fell back upon the only defenses available to her, one of these being to seek compensation in other realms as public approval became a substitute for lost parental love (Butscher, 2003). Her drive for success was an emulation of her deceased father’s own discipline and ambition, and this drive fuelled Sylvia’s poetic talent (Butscher, 2003; Rose, 2013). According to Schwartz’s model, her search for meaning through her poetry served as the vehicle through which her firefighters could dissociate from the pain of her exiled, wounded child parts (Panelatti, 2018).

Sylvia’s first published poem appeared in the Boston Sunday Herald only 6 months after her father’s death (Butscher, 2003; Wilson, 2013). Her drive for success and fame thus emerged in her childhood and, although she never specifically mentioned Otto’s death, she was quite aware of the link between her childhood artistic efforts and that traumatic event (Butscher, 2003; Rose, 2013). Even her intense fascination with the ocean extended to her father, whom she described as a Neptune-like character serving as a “father-sea-god muse” (Plath, 2000, p. 399). According to Rollyson (2013), poetry proved to be a mediating point between Sylvia and the world, a conjoining like that of land and sea. To Sylvia, words in poetry gave meaning to her world. They made her want to cry, but they also made her very happy. “Poetry had that power over her. She would live and die by it” (Rollyson, 2013, p. 15).

Sylvia’s poetry emanated from her storytelling parts and these parts titrated the influence of her vulnerable, exiled parts, thereby allowing the managerial parts of her system to cope with the overwhelming grief of her father’s tragedy. The trauma of his loss added even more injury to Sylvia’s already injured inner-child parts and reinforced the imprisonment of her exiled parts. Although the unrelentless imprisonment of Sylvia’s exiles trapped her in a world of darkness, this darkness also catalysed her efforts to escape the prison of her emotional suffering. Her emotional suffering fuelled her search for meaning and words and poetry proved to be her most potent weapons in her quest to create a meaningful life (Panelatti, 2018).

### The Anguish of the Exiles during Adolescence (1945–1952)

Although the end of World War II heralded a period of extreme optimism for society, for Sylvia it marked the commencement of an adolescent period characterised by self-consciousness, inner turmoil, harmful behaviour and identity confusion. At the age of 13, Sylvia began to keep a dream book in which she recorded her monstrous nightmares. She used the recorded nightmares as inspiration to write gothic mystery stories (Wilson, 2013). These stories provided an outlet through which she could express some of the darkness and morbidity that she hid behind her outer mask of normality and perfect amiability. It is speculated that Sylvia’s monstrous nightmares were manifestations of the desperation of her exiles and the severity of pain with which they were burdened. Sylvia’s journal entries also bore testimony to the severity of pain with which her exiles were burdened, as manifested in her imagery of herself drowning in a sea of negative emotions, including fear, envy, doubt, self-hate and madness. The extent to which Sylvia’s exiles were burdened could be associated with her dependency on her mother at this stage of her development. Not only was Sylvia financially dependent on her mother, she also had to share a room with her and she became so emotionally dependent on her, that she came to regard her mother as an extension of herself, rather than as a separate person.

Middle adolescence marked the period around Plath’s entry into senior high school. At the time, the United States of America was characterised by its complacent attitude of conformity and consumerism. Nonetheless, there was a pervasive feeling of uncertainty due to the unsettling effects of McCarthyism (Gill, 2008) on the country’s politics, and Sylvia was profoundly affected by the country’s seeming indifference to the Cold War and the Korean War.

Sylvia’s tendency to write cruel comments about those close to her, and projecting onto others, fantasies, wishes and motivations which seldom had bearing on reality, could have been the result of a highly polarised IFS. The polarised position of Sylvia’s protective parts could have contributed to the discrepancy between reality and Sylvia’s imaginary world, as well as to her obscured insight into her own reality. Although Aurelia was a very encouraging and self-sacrificing parent, she raised her children with the knowledge that there was a correlation between good behaviour and love, and she enforced a strict moral regime through martyrdom to principles and values. In terms of the IFS model, Aurelia’s parts-led parenting style created burdens for Sylvia, which polarised both the external family system and Sylvia’s IFS. The polarisation obstructed the emergence of Sylvia’s Self, thereby preventing the clarity of insight required for the establishment of a clear identity. Sylvia’s experience of herself as being fragmented into many different and often conflicting identities emanated from a severely polarised IFS which prevented the sense of continuity and integration required for effective Self-leadership. The intensity of behaviour of Sylvia’s protectors is attributed to the extent of pain experienced by her exiles, and would explain why her only recourse to some semblance of Self-led leadership was through her imagination, where the Self had “supernatural powers” (Butscher, 2003, p. 14).

Sylvia’s later adolescence was marked by her entry into junior college. Although the Cold War period following World War II was a time of peace and relative abundance, it was also a time of extreme anxiety and uncertainty, which greatly influenced Sylvia’s cultural and literary milieu. Aurelia’s staunch Calvinistic principles of religious and social propriety, and the stifling, hypocritical standards of Sylvia’s socio-cultural milieu, could have aggravated the polarisation of her IFS. Sylvia’s adolescent compulsions, especially for food and sexual activity, could be attributed to the polarised struggle between her managers and firefighters as they sought to control the emotional pain of her exiles. The physical manifestation of Sylvia’s depressive episodes whenever she was plagued by sinusitis and menstrual cramps, could also have emanated from the conflict between her polarised parts. Sylvia’s writing served as a meaning-making tool through which she could dissociate from the painful emotions that burdened her exiles (Panelatti, 2018).

### The Self’s Search for Meaning during Adulthood (1953–1963)

This historical period, which marked Plath’s lifespan between 21 and 23 years, commenced with Sylvia’s editorial internship in New York and coincided with her senior college years at Smith College. Although the outside world may have perceived her internship as the pinnacle of her literary achievement, and the epitome of the American Dream, to Sylvia the month in New York represented a traumatic time of progressive deterioration and ever-increasing despair.

Sylvia’s escalating feelings of failure and inferiority, despite all her accomplishments, were the result of her over-burdened system which prevented access to the healing benefits of Self-leadership. Sylvia’s sense of purposelessness when she returned from her guest-editorship in New York could have emanated from her lack of Self-leadership. Sylvia’s anguish of keeping the inner turmoil of her exiles securely locked away, whilst trying to meet her managers’ demands of efficiency, put so much pressure on her IFS, that her firefighters saw suicide as the only solution to saving her. The Electroconvulsive Therapy (ECT) to which Sylvia was subjected after she was diagnosed with depression, only served to increase the desperation of her exiled suicide parts, thereby intensifying her depression. The poorly administered electroshock treatments fractured and deleted Sylvia’s already splintered sense of identity and impaired her memory to such an extent that even joyful past experiences were replaced by cynicism and doubt. Added to this was the fact that the electroshock treatments aggravated Sylvia’s inability to sleep to the point that she developed chronic insomnia. Consequently her mental functions also began to deteriorate, and she felt incapable of the very activity which gave meaning to her life, namely writing (Wilson, 2013). In a letter she confessed her fear at the prospect of not being able to write again, believing that no-one would find her interesting if she lost this ability (Plath, 1992). In this state of despair, Sylvia began associating her depression with her deceased father. Overwhelmed by disturbing thoughts and emotions related to her father, suicide became not just a possibility, but a desired goal in Sylvia’s mind.

Sylvia’s suicide attempt of 1953 provides the most pertinent manifestation of the role of her firefighters during her senior college years. Her emotional pain induced an array of firefighting responses, including self-inflicted cuts, a drowning attempt, the abuse of sleeping pills and excessive sexual activity. Sylvia’s inability to establish a secure interpersonal relationship was aggravated by her experience of her father’s death as an abandonment. Her use of poetry as a vehicle to reify the lost father figure, could be attributed to the dissociative techniques of her firefighting protectors. Otto remained a constant force in Sylvia’s imagination and although she acknowledged, in her poem *The Colossus*, that she would never be able to get her father “put together entirely/pierced, glued, and properly jointed” (Plath, 2008, p. 12), she continued to recreate him in her works, using words as a substitute for his absence and the love she craved from him. In her diary, she admitted to being in search of “the gigantic paternal embrace of a mental colossus” (Plath, 2000, p. 163), believing that there was a strong link between the loss of her father and her compulsive desire to find a replacement for him in the men she dated (Plath, 2000).

Sylvia’s inability to establish secure interpersonal relationships characterised by genuine intimacy, spontaneity and warmth, emanated from a highly polarised IFS, which blocked her capacity to establish a heartfelt connection with her intimate partners. Although she harboured the dream of marrying a “demigod of a man,” she acknowledged the impossibility of that desire when she wrote: “I want a romantic non-existent hero” (Plath, 2000, p. 182). This realisation prompted her to retreat even further into her poetry and literature, where she often manufactured her own personal absolute of male perfection (Plath, 2000; Wilson, 2013).

In September 1955, Sylvia set sail for Europe where she would start a new life as a Fulbright scholar at Cambridge University in England. Sylvia’s dependency on her mother and her veneration of her lost father, could have prevented her from being Self-led and would have contributed to the overwhelming insecurity which she felt when she left North America to start a new life in England. Sylvia’s volatile relationship with Ted Hughes, whom she later married, provides the most pertinent manifestation of the desperation of her exiles during this historical period. Since Sylvia experienced her father’s death as a deliberate act of betrayal, her attraction to Ted could have been prompted by her exiles’ desperate search for a “redeemer” (Schwartz, 1995, p. 47). Sylvia’s failure to attain complete independence from her parents, could have prevented the unburdening of her IFS, resulting in emotional over-dependency on Ted and her impulsive decision to marry him. Her over-dependency on Ted, who served as a replacement for the lost father whom she still grieved, could have aggravated her exiles’ extreme experience of hopelessness and catalysed thoughts of suicide (Panelatti, 2018).

Sylvia’s contradictory and fluctuating emotions during this historical period, could be indicative of the polarised nature of her IFS. Her habit of escaping the pain that accompanied tragedy or loss by hiding in a fantasy world created by words, could be attributed to her firefighters and the measures taken by them to shield her from pain through the technique of dissociation. Her affinity for harrowing, adrenalin-filled incidents could also have been prompted by her firefighters in their quest to distract her system from the pain of exiled parts (Panelatti, 2018).

Plath’s lifespan between 25 and 27 years coincided with Sylvia and Ted’s return to the United States of America. The threat of nuclear war during the years of the Space Race fuelled the political tension at the time and greatly influenced Sylvia’s poetic voice and her confessional style of writing. Confessionalism proved to be a writing breakthrough for Sylvia since it allowed a deeper and more psychologically meaningful take on subject matter previously considered taboo. For someone like Sylvia, who was as attuned to the meaning of her inner reality as she was to the meaning of the socio-historical context in which she found herself, confessional writing proved to be the ideal vehicle to articulate personally-meaningful concerns, as well as concerns about the meaning of bigger political issues such as Hiroshima and the advent of the Cold War.

In terms of Schwartz’s IFS model, Sylvia’s exiles were frozen in circumstances of abuse and betrayal relating to her father. Her volatile, erratic and contradictory reactions to Ted’s infidelity and to his sadistic tendencies could thus be attributed to the inner battle between polarised perpetrator parts as they struggled to contain her exiled, wounded and traumatised parts. Sylvia’s atheism could have been transferred to her by her father, and this could have contributed to her involvement in occultish practices during this historical period (Panelatti, 2018). These practices could have formed part of the dissociative techniques implemented by her firefighters in their quest to douse the emotional flare-ups of her exiled parts.

Plath’s lifespan between 28 and 30 years coincided with Sylvia and Ted’s return to England. Although Sylvia’s surge of creativity after the births of her children could be attributed to the creative benefits of Self-leadership, it is more plausible to attribute it to the workings of her Self-like parts. Self-like parts do not have the Self’s ability to heal and could thus account for Sylvia’s incessant inner emotional turmoil, despite her efforts to maintain her mask of stability and perfect amiability. The death of a neighbouring friend affected Sylvia profoundly, not only because it activated exiled feelings related to her own father’s death, but also because Ted’s infidelity had intensified her exiled feelings of worthlessness and unlovability (Panelatti, 2018).

The most blatant manifestation of Sylvia’s highly polarised system and her protectors’ inability to keep her exiles contained, can be found in the final months of her life. In terms of Schwartz’s IFS model, the extreme measures implemented by Sylvia’s firefighters in these final months, attest to the severity of her internal crisis and the inability of her system to manage it. Sylvia’s preoccupation with black magic intensified after the break-up of her marriage, and could be indicative of the desperation of her firefighters. Her firefighters also prompted her to escape to the imaginary world of her literary works through dissociation. The extent of Sylvia’s inner turmoil and the desperation of her firefighters to keep her pain contained, also manifested in her decision to start smoking; her addiction to sleeping pills and in the somatic expression of her suicide parts in the form of influenza and high fever. Sylvia’s fluctuating emotions, her intermittent high fever, her exhaustion from waking at four in the morning to write poetry, and the unrelenting cold weather, could have contributed to Sylvia’s despair, and made her feel lonelier and more isolated than she had ever felt before. In this state of physical and mental depletion, Sylvia’s firefighters had little energy to protect her system from the danger posed by her volatile exiles. The feeling of hopelessness experienced by Sylvia’s exiles was so extreme, that it catalysed her suicide (Panelatti, 2018).

The energy that Sylvia put into her writing in the final months of her life supports the view that it was through words that Sylvia sought to unravel the meaning of her inner anguish, and it was through words that she sought to give meaning to her existence. Words were Sylvia’s most powerful tool, and charged words, in particular, were her tonic (Rollyson, 2013). In the same way that she had wielded words to win parental affection in childhood, identifying with the physicality of words to such an extent that they were her only comfort, so too she created her life and gave meaning to her existence through her words. In her poem *Kindness*, written days before her suicide, she declared her dependency on words when she said “The blood jet is poetry, / There is no stopping it” (Plath, 2008, p. 270).

One could say that the inscription on Sylvia’s tombstone—a quote from the Bhagavad Gita, which read: “Even amidst fierce flames the golden lotus can be planted” (Alexander, 1999, p. 332), was prophetic. Although Sylvia Plath fought with the negative parts of her makeup and lost the battle in the end, because the fierce flames of her exiled parts could not be extinguished, she used the passionate heat of those flames to breathe timeless meaning into her poetry, and for that, the legacy of her life and works blooms eternally.

### Conclusions and Recommendations

This article aimed to elucidate Plath’s meaning-making narratives, within her life’s puzzle of parts, by utilising the IFS model of Schwartz. Biographical material on Plath’s life, interpreted according to Schwartz’s (2001) model, indicated that each stage of her life was characterised by parts-led functioning as a result of transferred burdens, imperfect care-taking, anxiety and traumatic emotional experiences. This resulted in polarisation of her different parts, which blocked the healing energy of her Self and aggravated feelings of worthlessness, shame and guilt. The application of Schwartz’s (2001) model to Sylvia Plath’s life, highlighted the important role played by Plath’s socio-historical context and its impact on her development and her intrapsychic processes. The IFS model recognised that the conflict generated by a hypocritical society that supported male supremacy, challenged Sylvia’s optimal development and complicated the fulfilment of her roles as wife, mother and poetess. Despite these challenges, however, “Plath spread her wings, over and over, at a time when women were not supposed to fly” (Clark, 2020, p. xvii).

The study of an imminent figure of literary acclaim in feminist history could be of great value in our current society, which seeks to empower women, but simultaneously expects them to occupy multiple roles. Although Plath’s life was short-lived, the plethora of poems, short stories, letters and journal entries which were published after her death, attest to the fact that she succeeded in leaving a meaningful legacy, despite the despair and trauma which characterised her life. Although Plath’s life stages were characterised by unsuccessful integration of opposing parts and an ever-increasing sense of fragmentation, the success which she garnered as a writer and the wealth of meaningful narratives which she produced in such a short period of time, attest to the benefit which Schwartz’s theory holds for ingenious and creative artists who manage to achieve greatness despite their inability to achieve the Self-differentiation required for Self-leadership. This article thus contributes to the current body of knowledge on imminent individuals who achieve greatness despite personal and contextual setbacks.

Sylvia resented that a woman’s intellectual, emotional and physical ambitions were at odds with societal expectations (Alexander, 1999; Kirk, 2009; Plath, 2000). This article validates the important role played by one’s socio-historical context in shaping human development. Future research may benefit from explaining and describing Sylvia Plath’s lifespan development from feminist perspectives which address gender-related criticisms. This will provide an important foundation for a constructive feminist stance from which research can be expanded, so that the experiences of women can be better understood (Horst, 1995). This article affirms the researchers’ conviction that an examination of the lives of extraordinary women who used their creative genius to extract meaning and create meaningful narratives from socio-historical issues, continues to be a significant endeavour for future psychobiographical researchers.

Not only did Plath make sense of her own suffering through her works, she also created a platform through which future generations could create meaning from suffering and be empowered by it. Although her IFS was fragmented, her art and the meaning which she breathed into it, “was not to fall” (Hughes, 2004, p. xx), and continues to fuel a fire that lights the way forward.

## References

[r1] Alexander, I. E. (1990). *Personology: Method and content in personality assessment and psychobiography*. Durham, NC, USA: Duke University Press.

[r2] Alexander, P. (1999). *Rough magic: A biography of Sylvia Plath*. New York, NY, USA: Da Capo Press.

[r3] Butscher, E. (2003). *Sylvia Plath: Method and madness*. Tucson, AZ, USA: Schaffner Press.

[r4] Clark, H. (2020). *Red Comet: The short life and blazing art of Sylvia Plath*. London, United Kingdom: Jonathan Cape.

[r5] Earley, J. (2012). *Resolving inner conflict: Working through polarisation using internal family systems therapy*. Larkspur, CA, USA: Pattern System Books.

[r6] Fouché, J. P. (1999). *The life of Jan Christian Smuts: A psychobiographical study* (Unpublished doctoral dissertation). University of Port Elizabeth, Port Elizabeth, South Africa.

[r7] Gill, J. (2008). *The Cambridge introduction to Sylvia Plath*. Cambridge, United Kingdom: Cambridge University Press.

[r8] Goulding, R. A., & Schwartz, R. C. (1995). *The mosaic mind: Empowering the tormented selves of child abuse survivors*. New York, NY, USA: W. W. Norton.

[r9] Green E. J. (2008). Individuals in conflict: An internal family systems approach. *The Family Journal: Counseling and Therapy for Couples and Families*, 16(2), 125–131. 10.1177/1066480707313789

[r10] Haddock S. A. Weiler L. M. Trump L. J. Henry K. L. (2017). The efficacy of internal family systems therapy in the treatment of depression among female college students: A pilot study. *Journal of Marital and Family Therapy*, 43(1), 131–144. 10.1111/jmft.12184 27500908

[r11] Horst E. A. (1995). Reexamining gender issues in Erikson’s stages of identity and intimacy. *Journal of Counseling and Development*, 73(3), 271–278. 10.1002/j.1556-666.1995.tb01748.x

[r12] Hughes, F. (2004). *“Foreward,” Ariel: The restored edition*. New York, NY, USA: HarperCollins.

[r13] Kirk, C. A. (2009). *Sylvia Plath: A biography*. New York, NY, USA: Prometheus Books.

[r14] Kumlu E. (2011). The Mona Lisa smile of Sylvia Plath: Destroying the distorted picture of reality. *Selçuk Üniversitesi Sosyal Bilimler Enstitüsü Dergisi* [*Selcuk University Social Sciences Institute Journal*], 25, 173–182.

[r36] Panelatti, A. F. (2018). *Sylvia Plath: A psychobiographical study* (Unpublished doctoral dissertation). University of the Free State, Bloemfontein, South Africa.

[r15] Perloff, M. G. (1979). Sylvia Plath’s “sivvy” poems: A portrait of the poet as daughter. In G. Lane (Ed.), *Sylvia Plath: New views on the poetry* (pp. 155–77). Baltimore, MD, USA: John Hopkins University Press.

[r16] Plath, A. S. (1992). *Letters home by Sylvia Plath: Correspondence 1950–1963*. New York, NY, USA: Harper & Row.

[r17] Plath, S. (1966). *The Bell Jar*. London, United Kingdom: Faber and Faber.

[r18] Plath, S. (2000). *The unabridged journals of Sylvia Plath, 1950–1962*. New York, NY, USA: Random House.

[r19] Plath, S. (2004). *Ariel: The restored edition*. New York, NY, USA: Harper Collins.

[r20] Plath, S. (2008). *The Colossus*. London, United Kingdom: Faber & Faber.

[r21] Ponterotto J. G. Reynolds J. D. (2017). Ethical and legal considerations in psychobiography. *American Psychologist*, 72(5), 446–458. 10.1037/amp0000047 28726453

[r22] Rollyson, C. (2013). *American Isis: The life and art of Sylvia Plath*. New York, NY, USA: St. Martin’s Press.

[r23] Rose, J. (2013). *The haunting of Sylvia Plath*. London, United Kingdom: Virago Press.

[r24] Schwartz, R. (1995). *Internal family systems therapy*. New York, NY, USA: Guilford Press.

[r25] Schwartz R. C. (1987). Our multiple selves. *Family Therapy Networker*, 11, 24–31. 10.1007/978-3-319-15877-8_927-1

[r26] Schwartz, R. C. (2001). *Introduction to the internal family systems model*. Oak Park, IL, USA: Trailheads Publications.

[r27] Schwartz. R. C. (2003). The critical parts of me. In J. Kottler & J. Carlson (Eds.), *Bad therapy: Master therapists share their worst failures* (pp. 49–55). New York, NY, USA: Brunner-Routledge.

[r28] Schwartz R. C. (2013a). Moving from acceptance toward transformation with internal family systems therapy (IFS). *Journal of Clinical Psychology*, 69(8), 805–816. 10.1002/jclp.22016 23813465

[r29] Schwartz, R. C. (2013b). Depathologizing the borderline client. *Psychotherapy Networker*, 37, 32–39.

[r30] Sweezy, M., & Ziskind, E. L. (2013). *Internal family systems therapy: New dimensions*. New York, NY, USA: Routledge.

[r31] Twombly, J. H., & Schwartz, R. C. (2008). The integration of the internal family systems model and EMDR. In C. Forgash & M. Copeley (Eds.), *Healing the heart of trauma and dissociation with EMDR and ego state therapy* (pp. 295–311). New York, NY, USA: Springer.

[r32] Watzlawick, P., Weakland, J. H., & Fisch, R. (1974). *Change: Principles of problem formation and problem resolution*. New York, NY, USA: Jason Aronson.

[r33] Wilson, A. (2013). *Mad girl's love song: Sylvia Plath and life before Ted*. London, United Kingdom: Simon & Schuster.

[r34] Yin, R. K. (2009). *Case study research: Design and methods* (4th ed.). Thousand Oaks, CA, USA: Sage.

[r35] Young J. L. Collins B. M. (2018). For whose benefit? Comment on the psychobiography special section (2017). *American Psychologist Journal*, 73(3), 286–287. 10.1037/amp0000234 29648846

